# Supplementary material for: Adjuvanted influenza vaccination increases pre-existing H5N1 cross-reactive antibodies
Source: Nat Commun. 2026 Jan 7;17:1386. doi: 10.1038/s41467-025-68137-x (PMC12876951; doi:10.1038/s41467-025-68137-x)
Supplement: Supplementary file 1 — Supplementary Information [file 41467_2025_68137_MOESM1_ESM.pdf]

Supplementary information

Supplementary Table 1: Recombinant protein panel

Antigen	Strain	Recombinant protein expression	Sinobiological (Cat.No.)	Bead region
H1 trimer	A/California/07/2009	ExpiCHO (EPFL)	n/a	22
H5 trimer	A/Pelican/Bern/1/2022	ExpiCHO (EPFL)	n/a	12
Group 1 HA stalk	H6 head domain: A/mallard/Sweden/81/02 (H6N1) H1 stalk domain: A/California/04/09 (pandemic H1N1)	Baculovirus-Insect cells	n/a	13
Group 2 HA stalk	H7 head domain: A/mallard/Alberta/24/01 (H7N3) H3 stalk domain: A/Hong Kong/4801/2014 (H3N2).	Baculovirus-Insect cells	n/a	15
Human N1	A/California/04/2009	Baculovirus-Insect cells	Cat: 11058-V08B	26
Avian N1	A/Anhui/1/2005	Baculovirus-Insect cells	Cat: 11676-V08B	33

Supplementary Figures

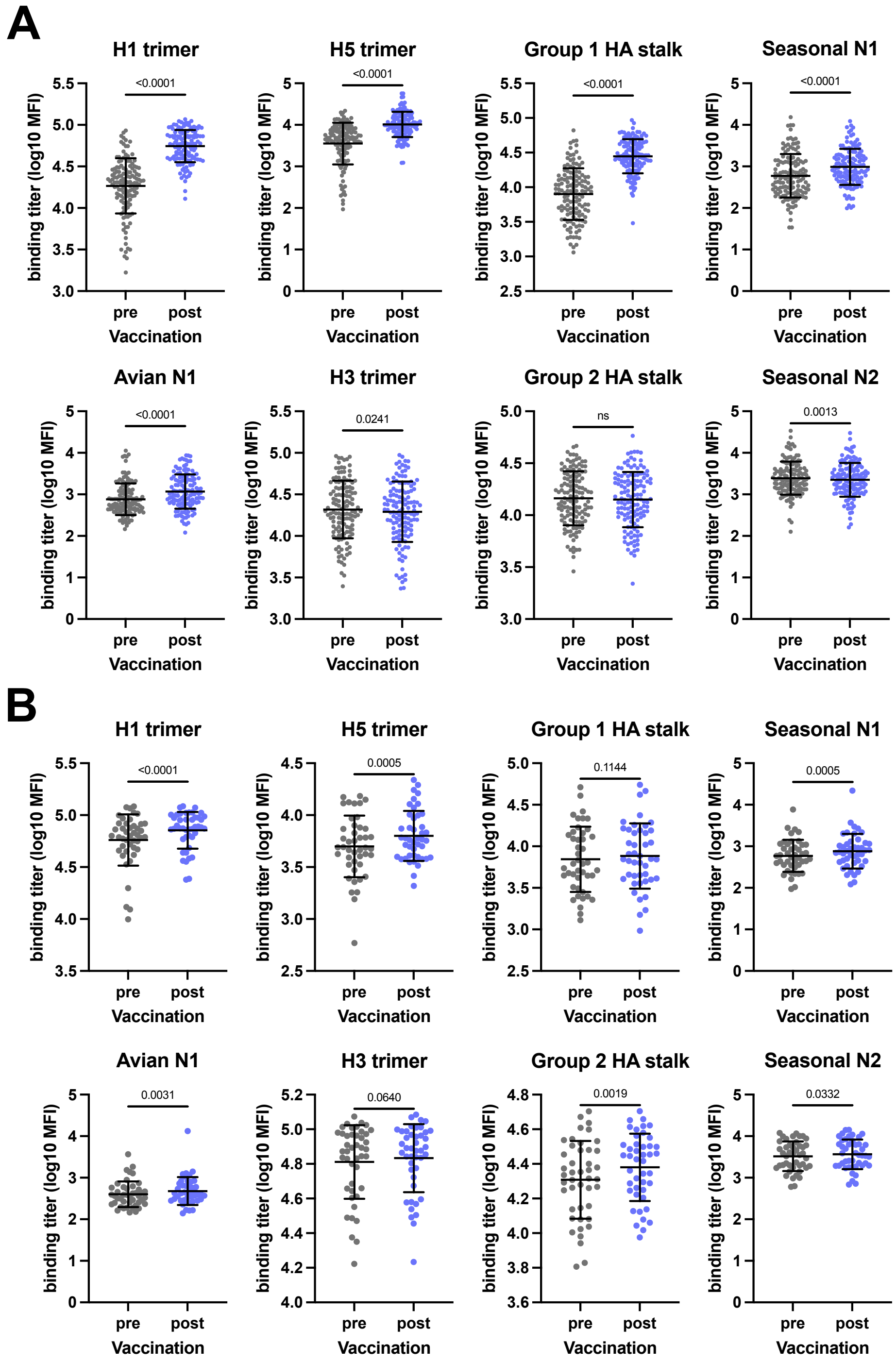

Supplementary figure 1: Pre- and post-vaccination antibody levels IgG antibody levels in median fluorescence intensity (MFI) units against influenza virus derived antigens in the 2009 (n=133) (**A**) and 2023 (n=44) (**B**) cohort. All antibody titres have been log10-transformed. Mean and SD is shown. Two-tailed Wilcoxon matched-pairs signed rank test was performed to compare pre and post vaccination antibody titres. Source data are provided as a Source Data file.

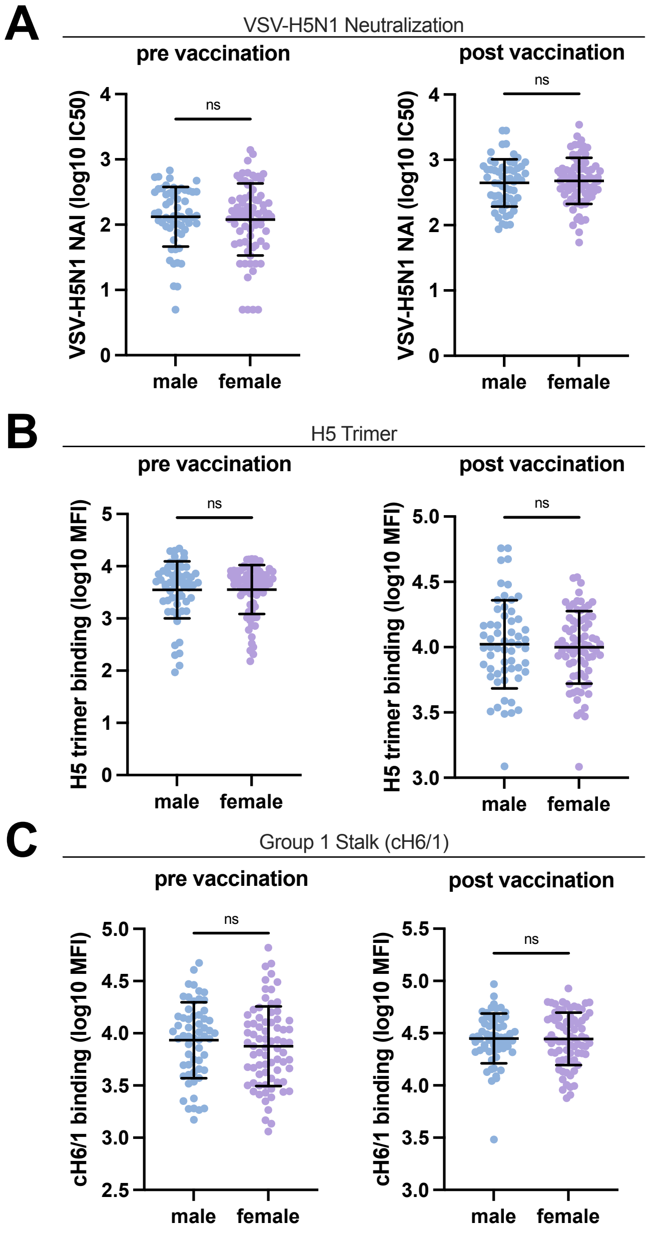

Supplementary figure 2: Antibody levels by sex. Sex-based differences in antibody responses in n=133 healthy adults (75 female, 58 male) pre and post pH1N1/AS03 vaccination. (**A**) VSV-H5N1 pseudovirus (A/Pelican/Bern/01/2022) cross-neutralizing antibody titres, (**B**) trimeric H5 HA and (**C**) group 1 stalk cH6/1 IgG antibody titres. All antibody titres have been log10-transformed. Mean and SD is shown. Statistical comparisons were performed using Mann-Whitney test (two-tailed). Source data are provided as a Source Data file.

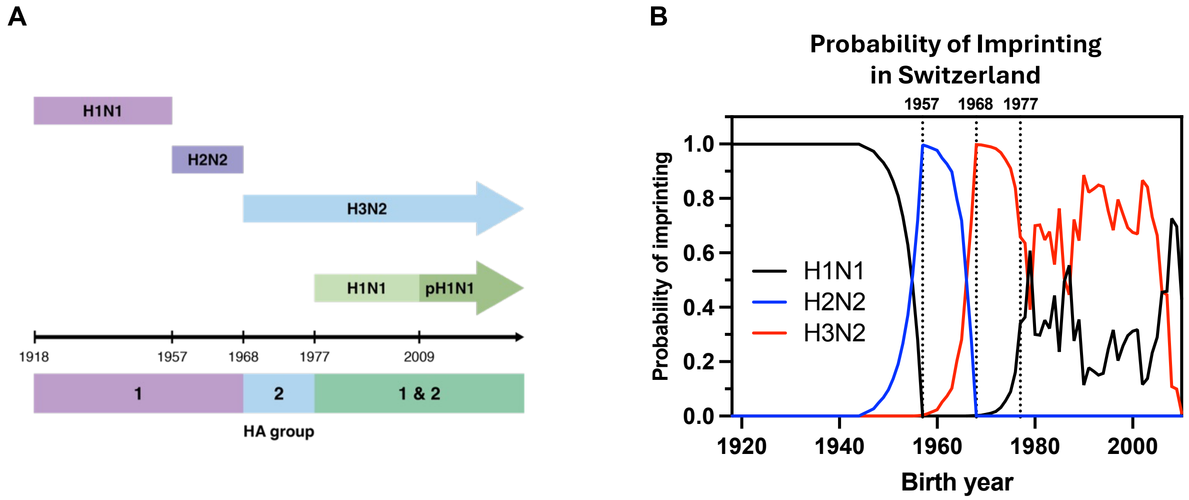

Supplementary figure 3: Influenza immune imprinting Influenza A virus circulation in the human population since 1918. **(A)** Group 1 HA influenza subtypes (H1N1 and H2N2) circulated between 1918 and 1968. Group 2 HA influenza subtype (H3N2) circulated between 1968-1977. After 1977, group 1 and 2 HA influenza subtypes (H1N1 and H3N2) co-circulate in the human population. **(B)** The probability of imprinting with H1N1 (black), H2N2 (blue) or H3N2 (red) for individuals based on their year of birth. Immune imprinting probabilities to influenza subtypes based on birth year in Switzerland between 1918 and 2010 were generated using methods described in Gostic et al. and the imprinting R package.

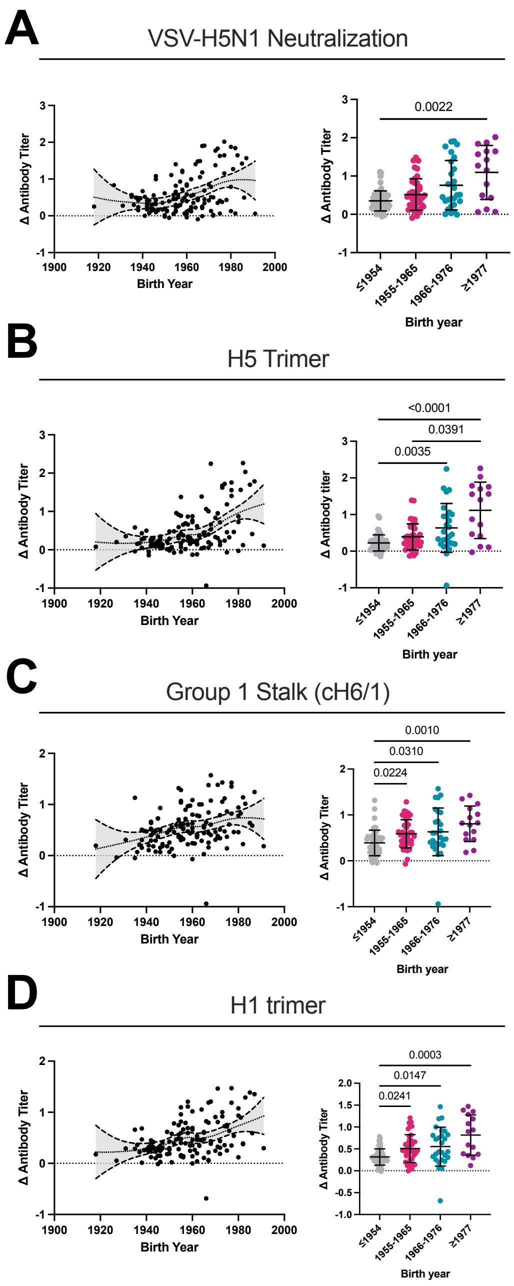

Supplementary figure 4: Effect of immune imprinting on increase of H5N1 cross-reactive antibody responses delta of pre- and post-vaccination (pH1N1/AS03) log10-transformed IgG antibody titers for (**A**) VSV-H5N1 pseudovirus (A/Pelican/Bern/01/2022) cross-neutralizing antibodies (**B**) trimeric H5 HA, (**C**) group 1 stalk cH6/1 and (**D**) trimeric H1 HA. Left panels: the solid lines represent the fitted curve calculated using the LOESS method. Dotted line represents 95% confidence bands of the best-fit line. Right panels: birth year cohorts are defined by imprinting probabilities. ≤1954 imprinted most likely with H1N1 (n=51), 1955-65 imprinted most likely with H2N2 (n=40), 1966-76 imprinted most likely with H3N2 (n=27), ≥1977 imprinted either with H1N1 or H3N2 (n=15). Mean and SD is shown. Kruskal-Wallis test (two-tailed) with Dunn's test were performed to compare the mean rank of each column and to correct for multiple comparisons. Source data are provided as a Source Data file.

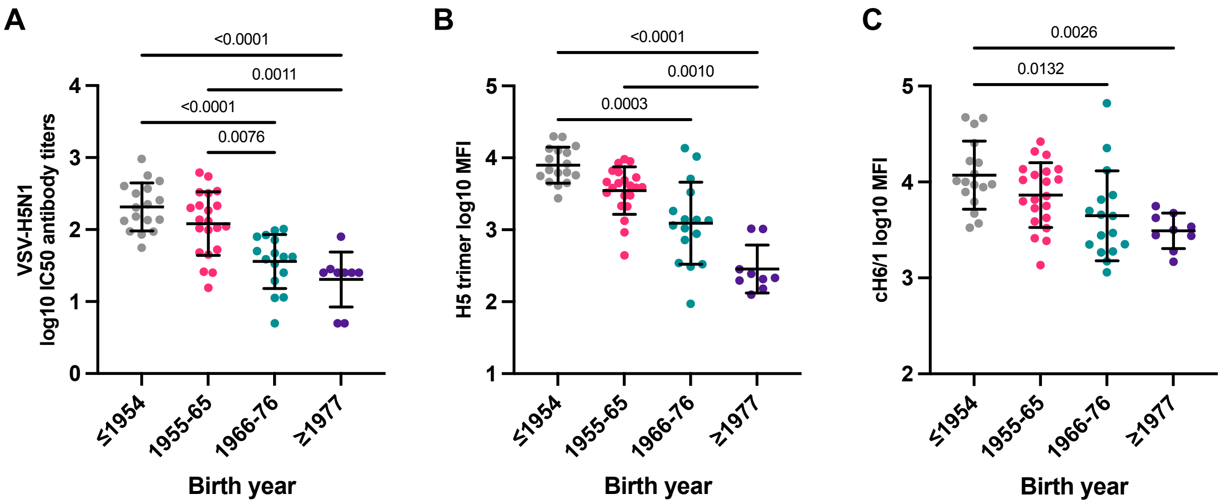

Supplementary figure 5: Immune imprinting in participants without previous influenza vaccination Effect of immune imprinting on H5N1 cross-reactive antibody responses before pH1N1/AS03 vaccination in individuals that have not received a seasonal influenza vaccination in 2008 or 2009. (**A**) VSV-H5N1 pseudovirus (A/Pelican/Bern/01/2022) cross-neutralizing antibody titres, (**B**) trimeric H5 HA and (**C**) group 1 stalk cH6/1. Birth year cohorts are defined by imprinting probabilities. ≤1954 imprinted most likely with H1N1 (n=17), 1955-65 imprinted most likely with H2N2 (n=21), 1966-76 imprinted most likely with H3N2 (n=16), ≥1977 imprinted either with H1N1 or H3N2 (n=9). Mean and SD is shown. Kruskal-Wallis test (two-tailed) with Dunn's test were performed to compare the mean rank of each column and to correct for multiple comparisons. Source data are provided as a Source Data file.
